# Are Lipid Profiles in Middle Age Associated with Famine Exposure during Prenatal and Early Postnatal Period?

**DOI:** 10.3390/nu12082266

**Published:** 2020-07-29

**Authors:** Xin-Yue Ding, Zhen-Yu Yang, Li-Yun Zhao, Wen-Hua Zhao

**Affiliations:** National Institute for Nutrition and Health, Chinese Center for Disease Control and Prevention, 27 Nanwei Road, Xicheng District, Beijing 100050, China; dxyany@hotmail.com (X.-Y.D.); yangzy@ninh.chinacdc.cn (Z.-Y.Y.); zhaoly@ninh.chinacdc.cn (L.-Y.Z.)

**Keywords:** lipid profile, undernutrition, early life, the Great Chinese Famine

## Abstract

Background: Undernutrition during early life may increase the risk of chronic diseases in adulthood, including dyslipidemia. Few investigations have confirmed the relationship between early life undernutrition and dyslipidemia in adulthood in China. Objectives: To assess the relationship between the Great Chinese Famine exposure during prenatal period or early postnatal period and lipid profiles in adulthood. Design: Data were extracted from the China Nutrition and Health Survey (CNHS) in 2010–2012, which included the participants who experienced the Great Chinese Famine during early life. Results: Participants who experienced the Great Chinese Famine in early postnatal period had a significantly higher prevalence of elevated total cholesterol (TC) (odds ratio: 1.60; 95% CI: 1.27, 2.02) than unexposed participants. Female (odds ratio: 1.71; 95% CI: 1.27, 2.31) were high risk than male (odds ratio: 1.46; 95% CI: 1.01, 2.11) and physical inactivity group (odds ratio: 1.65; 95% CI: 1.18, 2.29) were high risk than adequate physical activity group (odds ratio: 1.58; 95% CI: 1.21, 2.07). Similar effect of famine exposure on elevated low-density lipoprotein cholesterol (LDL-C) was observed, except that no significant difference was found between adequate physical activity group and physical inactivity group. Participants who experienced the Great Chinese Famine in prenatal period had a significantly higher prevalence of lowed high-density lipoprotein cholesterol (HDL-C) (odds ratio: 1.19; 95% CI: 1.03, 1.37) than unexposed. Female were more likely to have lower HDL-C (odds ratio: 1.44; 95% CI: 1.18, 1.74), but not found in male. Participants with physical inactivity were more likely to have lower HDL-C (odds ratio: 1.28; 95% CI: 1.02, 1.61), but not found in adequate physical activity group. Conclusions: People who experienced the Great Chinese Famine during early life, especially in females and people physical inactivity, would impair of lipid profiles in later life. Healthy lifestyle like adequate physical activity may partially alleviate the adverse effects.

## 1. Introduction

Undernutrition during early life may increase the risk of chronic diseases in adulthood [1,2,3,4], including dyslipidemia [5]. In 1965, Snapper first reported that severe undernutrition may cause alterations in the lipid profile according to studies on adults who had experienced early life undernutrition during World War II [6]. Some animal studies through guinea pig and rat observations have also suggested that manipulations of people dietary intake during early life may permanently alter cholesterol synthesis and plasma cholesterol concentrations [7,8,9]. These observations in animal models indicated that early childhood undernutrition may predispose to hypercholesterolemia and metabolic disorders directly through an interaction with cholesterol metabolism and indirectly by influencing lifestyle choices [10,11]. Previous studies focused on the association between early life exposure to undernutrition and dyslipidemia of adults in developed countries [5,12,13], but fewer relative studies were carried out in China [14]. All of the investigation subjects were survived from the Dutch famine and only confirmed the relationship between exposed to the famine in utero during late, mid or early prenatal and dyslipidemia of adults, however, none of the investigations were in China or confirmed the relationship between exposed to the famine in prenatal or early postnatal and dyslipidemia of adults.

Dyslipidemia is an important risk factor for cardiovascular diseases, including coronary heart diseases, stroke and atrial fibrillation [15,16,17]. The main indicators for evaluating dyslipidemia included total cholesterol (TC), triglyceride (TG), high-density lipoprotein cholesterol (HDL-C) and low-density lipoprotein cholesterol (LDL-C). Lately, studies have shown that the ratio of lipid profiles may reflect the relationship between lipid profiles and health, such as the ratio of total cholesterol (TC) to HDL cholesterol (TC: HDL cholesterol), the ratio of triglyceride (TG) to HDL cholesterol (TG: HDL cholesterol) and atherosclerosis index (AI, (TC-HDL-C)/HDL-C) [18,19,20,21,22].

The aim of this study was to analyze the relationship between famine exposure during prenatal and the early postnatal period and dyslipidemia in Chinese through studying The Great Chinese Famine lasting from 1959 to 1961.

## 2. Subjects and Methods

### 2.1. Participants

Study subjects were from the China Nutrition and Health Survey (CNHS) in 2010–2012, a national representative survey [23]. A stratified, multistage probability cluster sampling design was used in this survey, which has been described in detail previously [23]. In each survey county, 6 villages/communities were samples, in which 75 households were sampled and all inhabitants in these households were surveyed. The number of people at each survey county was not less than 1000. From the survey, study subjects born between 1 October 1959 and 30 September 1964, were selected for the current study (Table 1). To minimize misclassification of the exposure periods, participants date of birth between 1 October 1961 and 30 September 1962, were excluded because the exact dates of the start and the end of the food shortages caused by Chinese famine were not available in different regions [24]. Some 682 adults from Tibet and Hainan were excluded due to the difficulties in fingering out their severity of famine as well as 3204 adults due to the lack of their lipid profile information. All procedures involving participants were approved by the Medical Ethics Committee at the National Institute for Nutrition and Health, Chinese Center for Disease Control and Prevention (Ethic committee approval code: 2013–018). All participants provided their written informed consent.

### 2.2. Exposure Measurement

The exposure status of subjects was determined according to their dates of birth (Table 1). Severity of famine varied among different provinces due to local policies pertaining to food shortage, population density and weather conditions [25]. The severity of the famine was determined based on the excess death rate (EDR) of each province during the Great Chinese Famine [25]. Participants were classified into severely famine exposed group, moderately famine exposed group and lightly famine exposed group based on residential provinces and excluding participants who without local permanent residency. The EDR was calculated as the percentage change in mortality rate from the mean level in 1956–1958 to the highest value during the period 1959–1961 [25]. The EDR was divided into tertiles. Provinces with an EDR above the 66th percentile of the EDR were categorized as severely famine exposed region, equal to or below the 33rd of the EDR were categorized as lightly famine exposed region and otherwise as moderately famine exposed region.

### 2.3. Data Collection

Basic information such as age, gender and education, marital status, family income and physical activity was collected using a structured questionnaire. Anthropometric measurements included body height, weight and waist circumference. Height was measured using a stadiometer (model no. SG-210, Nantong Yue kin cervix equipment Co., Ltd., Nantong, China). Body weight was measured using a beam scale, which is model no. Both height and weight were measured without shoes. RGT-14-RT (Wuxi Weighing Factory Co., Ltd., Wuxi, China) [26]. Waist circumference was measured using a waist circumference tape, which is model no.0403 (Nanjing Kongki Commodity Co., Ltd., Nanjing, China) [26]. The accuracy of the height, weight and waist circumference measurements was 0.1 cm, 0.1 kg and 0.1 cm, respectively [26]. The anthropometric measurements methods is consistent with the standard anthropometric measurement methods in health surveillance [26]. TC measured by cholesterol oxidase aminoantipyrine phenol method (CHOD-PAP) (automatic biochemical analyzer), TG measured by phosphoglycerol oxidase 4-chloro acid method (automatic biochemical analyzer), HDL-C measured by direct method (automatic biochemical analyzer), but the measuring instruments of each survey point are not required to be uniform. The LDL cholesterol was calculated from measured TC, triglycerides and HDL cholesterol according to the Friedewald formula [27]. The classifications from the 2016 Chinese guideline was used for the management of dyslipidemia in adults [28] to define elevated TC, marginally elevated TC, low HDL-C, elevated LDL-C, marginally elevated LDL-C, elevated TG and marginally elevated TG (Table 2). Either elevated TC, low HDL-C, elevated LDL-C or elevated TG was defined as dyslipidemia. Marginally elevated TC and low HDL-C and marginally elevated LDL-C and marginally elevated TG meeting one or more can be define as marginal dyslipidemia. Either elevated TG or low HDL-C was defined as abnormal TG or HDL (hereinafter referred to as ANTH).

Working strength was categorized into lightly level and moderate to vigorous level. Exercise level was categorized into active and inactive. Moderate to vigorous physical activity (MVPA) less than 150 min per week and vigorous physical activity less than 75 min per week was defined as inactive level. Physical activity level was categorized into adequate physical activity and physical inactivity. Exercise level is inactive and working strength is lightly level was defined as physical inactivity, others was defined as adequate physical activity. Marital status was categorized into married, unmarried, divorced or widowed. Education level was categorized into below primary school, junior high school or below, high school or above. Per-capita annual income was categorized into Low (below 10,000 RMB), medium (10,000–29,999 RMB) and high (30,000 and above RMB). Height and weight were used to calculate BMI, by dividing weight (kg) by height squared (m^2^). Systemic obesity and abdominal obesity were defined using the Chinese criteria of weight for adults [29]. The BMI was calculated with normal weight, overweight and obesity. Normal weight was defined as BMI < 24 kg/m^2^. Overweight was defined as BMI ≥ 24 and < 28 kg/m^2^. Obesity was defined as BMI ≥ 28 kg/m^2^. Abdominal obesity was defined as waist circumference > 90 cm in men and ≥ 85 cm in women.

### 2.4. Statistical Methods

The SAS version 9.4 (SAS Institute, Inc., Cary, NC, USA) was used to analyze all statistical and the two-sided *p*-value < 0.05 was considered statistically significant. The variables TC, TG, TC-HDL-C ratio, TG-HDL-C ratio and AI had a skewed distribution and were logarithm transformed for further analysis. The results for these variables are given as geometric means and Standard Deviation (SD) and other variables are given as means and SD. Generalized linear models (GLM) was used to calculate the differences between the lipid profiles of unexposed subjects, exposed prenatal and exposed after born. First, we used GLM compared exposed groups with the unexposed group, adjusted for gender, age, obesity status, abdominal obesity or not, education level, marital status, per-capita annual income, exercise, working strength, control lipid plasma by drugs or others and severity of famine. Moreover, we stratified the analyses according to gender and severity of famine and physical activity. Second, we using the logistic regression model compared postnatal-exposed group and prenatal-exposed group to the unexposed group also adjusted for gender, age, obesity status, abdominal obesity or not, education level, marital status, per-capita annual income, exercise, working strength, control lipid plasma by drugs or others and severity of famine. Then we stratified the analyses according to gender, severity of famine and physical activity. The odds ratios (95% CI) were plotted in a graph using Stata 13.0. Sensitivity analysis was also performed in this study. Participants who used drugs or other means to control lipid profiles were excluded from this study as a performance of sensitivity analysis, to rule out the underestimation caused by the control of lipid profiles.

## 3. Results

In total, 9492 participants were included in the study: of this, 2068 were exposed to famine during the early postnatal period and 1592 were exposed to famine during prenatal. Participants who experienced the Great Chinese Famine in prenatal or postnatal period had a higher level of TC, HDL-C, LDL-C, TC/HDL-C, TG/HDL-C and AI. Mean TC level was higher in postnatal-exposed group and in prenatal-exposed group than in unexposed group, respectively 4.78 mmol/L vs. 4.67 mmol/L (*p* < 0.001), 4.73 mmol/L vs. 4.67 mmol/L (*p* = 0.017). The LDL-C value in postnatal-exposed group and in prenatal-exposed group was higher than unexposed, respectively 2.87 mmol/L vs. 2.79 mmol/L (*p* < 0.001), 2.85 mmol/L vs. 2.79 mmol/L (*p* = 0.007). The HDL-C value in postnatal-exposed group was higher than unexposed, but prenatal-exposed group lower than unexposed, respectively 1.20 mmol/L vs. 1.18 mmol/L (*p* = 0.028), 1.16 mmol/L vs. 1.18 mmol/L (*p* = 0.052). The TC/HDL-C value in prenatal-exposed group was higher than unexposed, about 4.40 vs. 4.23 (*p* < 0.001). The TG/HDL-C value in prenatal-exposed group was higher than unexposed, about 1.70 vs. 1.58 (*p* = 0.027). The AI value in prenatal-exposed group was higher than unexposed, about 3.40 vs. 3.23 (*p* < 0.001) (Table 3).

Participants who experienced the Great Chinese Famine in postnatal were significantly associated with level of famine, age, education level, exercise level and working strength. Participants who experienced the Great Chinese Famine in prenatal were significantly associated with level of famine, age, education level and exercise level. This suggests that the association between participants who experienced the Great Chinese Famine in postnatal or in prenatal period and lipid profiles in adulthood was possibly confounded by the covariates, and it may be of importance to adjust for the covariates.

After adjusting for gender, age, education level, marital status, per-capita annual income, exercise, working strength and severity of famine, no significant differences TG level were observed in postnatal-exposed group or in prenatal-exposed group compared to unexposed group.

TC level was lower in participants prenatal-exposed than participants unexposed (4.48 mmol/L vs. 4.62 mmol/L *p* = 0.034), these results were not significant in postnatal-exposed group.

HDL-C level was lower in participants prenatal-exposed than participants unexposed (1.09 mmol/L vs. 1.14 mmol/L, *p* = 0.004), these results were not significant in postnatal-exposed group. Female, lightly exposed group and physical inactivity group in prenatal-exposed group all had lower mean HDL-C level than participants unexposed, respectively (1.14 mmol/L vs. 1.2 mmol/L, *p* = 0.011; 1.14 mmol/L vs. 1.21 mmol/L, *p* = 0.006; 1.06 mmol/L vs. 1.13 mmol/L, *p* = 0.004).

LDL-C level was lower in participants postnatal-exposed in severely exposed group than participants unexposed (2.39 mmol/L vs. 2.58 mmol/L, *p* = 0.034). However, this effect was not significant in prenatal-exposed group.

TC/HDL-C, TG/HDL-C and AI were higher in participants prenatal-exposed in physical inactivity group than participants unexposed, respectively (4.53 vs. 4.26, *p* = 0.01; 1.63 vs. 1.42, *p* = 0.033; 3.46 vs. 3.19, *p* = 0.01). However, this effect was not significant in postnatal-exposed group.

### Dyslipidemia

**Figure 1:**
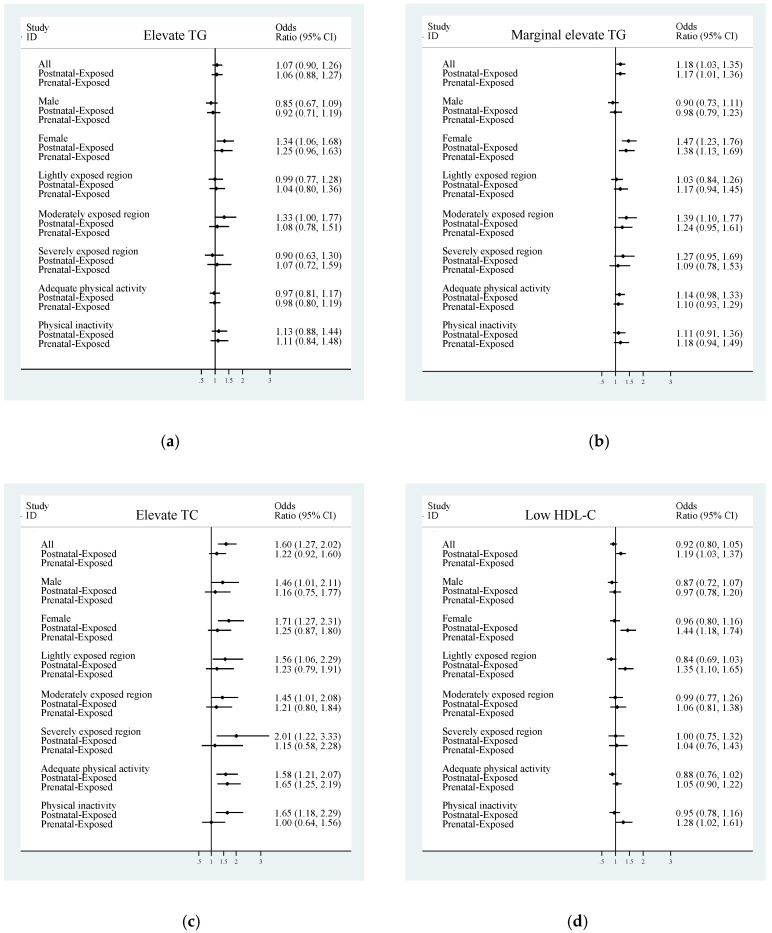

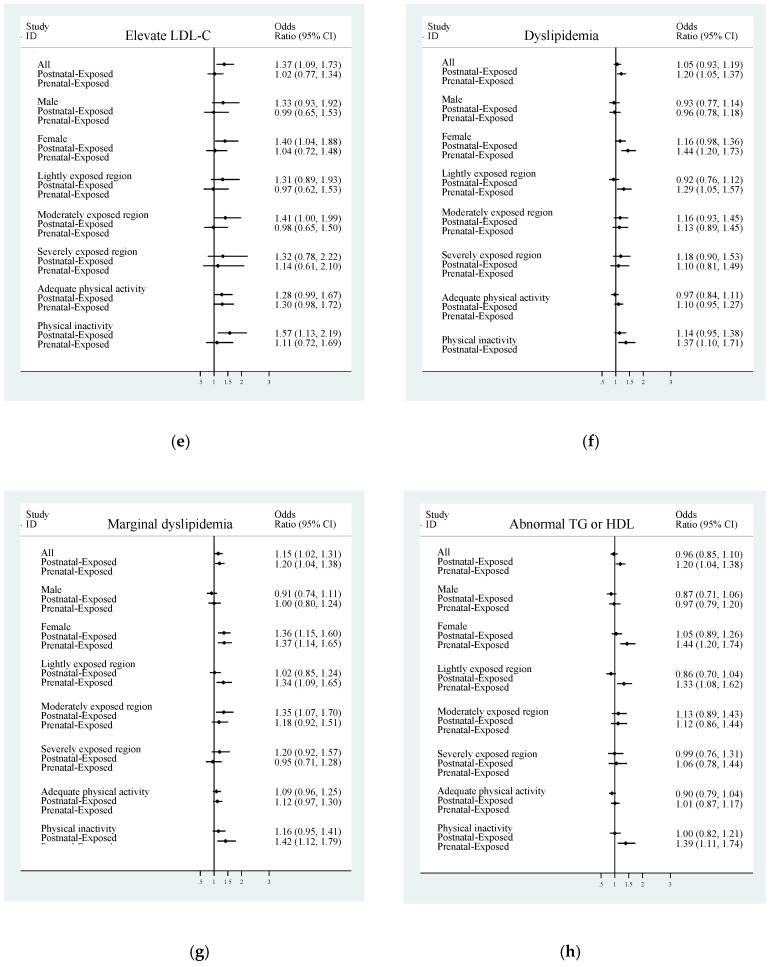
Odds ratio (OR) (and 95% CI), after adjustment, among participants postnatal-exposed, prenatal-exposed and unexposed participants. (**a**) with elevated TG; (**b**) in marginally elevated TG; (**c**) with elevated TC; (**d**) with low HDL-C; (**e**) with elevated LDL-C; (**f**) with dyslipidemia; (**g**) with marginal dyslipidemia; (**h**) with abnormal TG or HDL.

After being adjusted, female in postnatal-exposed group were more likely to have elevated TG (odds ratio: 1.34; 95% CI: 1.06, 1.68) than unexposed group. However, this effect was not significant in prenatal-exposed group (Figure 1a).

Participants in postnatal-exposed group had a significantly higher prevalence of elevated TC (odds ratio: 1.60; 95% CI: 1.27, 2.02), compared to the unexposed group. Both male and female in postnatal-exposed group had a significantly higher prevalence of elevated TC than unexposed group with an odds ratio (95% CI) of 1.46 (1.01, 2.11) and 1.71 (1.27, 2.31). Participants in postnatal-exposed group and lived in severely famine exposure region had a significantly higher prevalence of elevated TC (odds ratio: 2.01; 95% CI: 1.22, 3.33) than unexposed group, the same holds true for who lived in lightly famine exposure region (odds ratio: 1.56; 95% CI: 1.06, 2.29) and who lived in moderately famine exposure region (odds ratio: 1.45; 95% CI: 1.01, 2.08). However, this effect was not significant for prenatal-exposed group. The prevalence of elevated TC of participants in postnatal-exposed group and with physical inactivity were significantly higher (odds ratio: 1.65; 95% CI: 1.18, 2.29) than unexposed group, these results were not significant in prenatal-exposed group. Participants who with adequate physical activity had a significantly higher prevalence of elevated TC, both in prenatal-exposed group (odds ratio: 1.65; 95% CI: 1.25, 2.19) and in postnatal-exposed group (odds ratio: 1.58; 95% CI: 1.21, 2.07) than in unexposed group (Figure 1c).

Participants in prenatal-exposed group had a significantly higher prevalence of lowed HDL-C (odds ratio: 1.19; 95% CI: 1.03, 1.37), compared to the unexposed group. Female were more likely to have lower HDL-C in prenatal-exposed group (odds ratio: 1.44; 95% CI: 1.18, 1.74) than unexposed group, but not found in male. In lightly famine exposure region, participants in prenatal-exposed group had a significantly higher prevalence of lowed HDL-C (odds ratio: 1.35; 95% CI: 1.1, 1.65) than unexposed group, but it was not found in moderately famine exposure region and in severely famine exposure region. Participants in prenatal-exposed group who with physical inactivity had a significantly higher prevalence of lowed HDL-C (odds ratio: 1.28; 95% CI: 1.02, 1.61) than unexposed group. However, this effect was not significant for postnatal-exposed group (Figure 1d).

Participants in postnatal-exposed group had a significantly higher prevalence of elevated LDL-C (odds ratio: 1.37; 95% CI: 1.09, 1.73), compared to the unexposed group. Female were more likely to have elevated LDL-C in postnatal-exposed group (odds ratio: 1.4; 95% CI: 1.04, 1.88) than unexposed group, but not found in male. Participants in postnatal-exposed group and with physical inactivity had a significantly higher prevalence of elevated LDL-C than unexposed group (odds ratio: 1.57; 95% CI: 1.13, 2.19), these results were not significant for participants who with adequate physical activity. However, this effect was not significant in prenatal-exposed group (Figure 1e).

Participants in prenatal-exposed group had a significantly higher prevalence of dyslipidemia (odds ratio: 1.2; 95% CI: 1.05, 1.37), compared to the unexposed group. Female were more likely to have dyslipidemia in prenatal-exposed group (odds ratio: 1.44; 95% CI: 1.2, 1.73) than unexposed group, but not found in male. Participants in prenatal-exposed group and lived in lightly famine exposure region had a significantly higher prevalence of dyslipidemia (odds ratio: 1.29; 95% CI: 1.05, 1.57) than unexposed group, these results were not significant in moderately famine exposure region and in severely famine exposure region. Participants in prenatal-exposed group and with physical inactivity had a significantly higher prevalence of dyslipidemia (odds ratio: 1.37; 95% CI: 1.1, 1.71) than unexposed group, but were not significant for participants who with adequate physical activity. However, this effect was not significant in postnatal-exposed group (Figure 1f).

Participants in prenatal-exposed group had a significantly higher prevalence of ANTH (odds ratio: 1.2; 95% CI: 1.04, 1.38), compared to the unexposed group. Female were more likely to have ANTH in prenatal-exposed group (odds ratio: 1.44; 95% CI: 1.2, 1.74) than unexposed group, but not found in male. Participants in prenatal-exposed group and lived in lightly famine exposure region had a significantly higher prevalence of ANTH (odds ratio: 1.33; 95% CI: 1.08, 1.62) than unexposed group, but these results were not significant in moderately famine exposure region and in severely famine exposure region. Participants in prenatal-exposed group and with physical inactivity had a significantly higher prevalence of ANTH (odds ratio: 1.39; 95% CI: 1.11, 1.74) than unexposed group, but were not significant for participants who with adequate physical activity. However, this effect was not significant in postnatal-exposed group (Figure 1h).

Then participants who controlled their lipid plasma using drugs or others means were excluded in sensitivity analyses. To rule out the underestimation that the relationship between famine exposure during early life and lipid profiles in adult, which caused by the artificial control of lipid profiles. Compared to unexposed group, participants in postnatal-exposed group and lived in severely famine exposure region and lived in moderately famine exposure region both appeared to a higher prevalence of marginally elevated TG (odds ratio: 1.35; 95% CI: 1.01, 1.81; odds ratio: 1.47; 95% CI: 1.03, 2.08, respectively). Female in postnatal-exposed group turned to no higher prevalence of elevated TC, the same holds true for participants who lived in lightly famine exposure region. No changes were observed of effect on others lipid profiles indicators in both participants in postnatal-exposed group and prenatal-exposed group.

## 4. Discussion

In this study, we found that those who experienced the Great Chinese Famine in the early postnatal period (postnatal-exposed group) would increase the risk of elevated TC, elevated LDL-C and marginal dyslipidemia in adulthood. Moreover, those who experienced the Great Chinese Famine in the prenatal period would increase the risk of low HDL-C, dyslipidemia, marginal dyslipidemia and abnormal TG or HDL in adulthood. Female were more vulnerable to the Great Chinese Famine exposure than male. Participants in prenatal-exposed group and lived in lightly famine exposure region were more vulnerable on low HDL-C and dyslipidemia. Physical inactivity exacerbated the Great Chinese Famine.

The mechanisms behind the association between famine exposure in early life and the risk of dyslipidemia in adult life was still not clear. The reason may be that alterations in fetal nutrition and endocrine status lead to developmental adaptations that permanently change the body structure, physiology and metabolism, thereby predisposing individuals to cardiovascular, metabolic and endocrine diseases in adult life [30,31,32], according to the Barker hypothesis. Studies suggested that the mechanisms underlying the effects of prenatal exposure to undernutrition may be mediated by the induction of leptin resistance [33,34,35] and changes in hypothalamic development [36], but it has not been clearly confirmed.

Unlike previous study, we add to the analysis of participants who experienced famine in the early postnatal period. Moreover, found it will increase the risk of elevated TC, elevated LDL-C and marginal dyslipidemia in adulthood. This means, not just in prenatal period, nutritional status in early childhood period also affects the health of lipid profiles in adulthood. However, we found participants who experienced the Great Chinese Famine in early postnatal period and in prenatal period had different effect on indicators. Participants who experienced famine in the prenatal period were more vulnerable than those who experienced famine in the early postnatal period, compared to the participants unexposed. The reason behind this result may be that the Chinese famine lasted for three years (1959–1961), which means participants who experienced famine in the prenatal period usually experienced famine in the early postnatal period in our study.

In our study, female, not male, who experienced the Great Chinese Famine during early life would affect the health of lipid profiles in later life. That is consistent with the previous studies [12]. That can be explained by two reasons. First, survival effects. The need of nutrition is higher for male babies to survive than female babies, which means female babies survived from famine more likely experienced the famine during early life than male babies [37] and male survivors may have “acceptable” nutrition exposure during early life. Second, gender discrimination. Male survivors may have better nutrition than female during famine, because of the son preference tradition in China [38]. Studies showed that undernutrition during early life had larger long-term impacts on the health of later life for females than for males [39,40,41].

Our study found participants who lived in lightly famine exposure region were more vulnerable on low HDL-C and dyslipidemia than who lived in moderately and severely famine exposure region. Survivor bias may be the main reason. Participants survived from moderately and severely famine exposure region generally had better physical constitution and more nutrition intake.

In our study, for the first time, we addition stratified by physical activity to analyze the relationship between the famine exposure in prenatal or early postnatal period and lipid profiles in adulthood period. We found people who experienced famine in early life and physical inactivity would be more susceptible to lipid profiles in adulthood. On the contrary, people with adequate physical activity may lessen or even eliminate this effect. This means ensuring adequate physical activity is an effective measure to keep the health of lipid profiles.

One of the limitations of the present study is lack of the birth weight data. Previous studies usually used birth weight to assess the nutritional status of people during prenatal. Instead of, we use the date of birth to estimate their exposure during the Great Chinese Famine, because of we cannot get the birth weight in the present study. However, some studies suggested the effect of exposure to famine in prenatal on adult lipid profiles may not be explained by differences in body size at birth [13], so this was not considered as a major limitation. Another limitation of the present study is the selection of control group. The Chinese famine affected almost the entire country, the unexposed group only can be classified by birth date rather than region. Moreover, one limitation is used the current resident provinces to estimate the severity of exposed from the Great Chinese Famine during 1959–1961, which may lead to bias. However, less people would change their resident provinces in China, given by the tradition culture. Moreover, we estimation the severity of exposed based on local (county) status of the famine severity because no individual famine exposure available. Despite these limitations, our research also had irreplaceable advantages. Our study used data from CNHS in 2010–2012, which is reliable and with detailed information regarding sociodemographic characteristics, lifestyle factors and birthplace, and we used more indicators to assess the health of lipid profiles than other studies. Therefore, our study for the first time estimated the relationship between experience the Great Chinese Famine during early life and lipid profiles in adulthood and provides direction for future research.

In conclusion, people who experienced the Great Chinese Famine During either in prenatal or early postnatal exposure, especially in females and people physical inactivity, would increase impacted the health of lipid profiles in later life. The present study shows that healthy lifestyle like adequate physical activity may partially alleviate the adverse effects.

## Figures and Tables

**Table 1:** Grouping of research objects and basis.

Group	Birth Date	N
Unexposed	1962.10.01–1964.09.30	5832
Exposure during early postnatal	1959.10.01–1960.09.30	2068
Exposure during prenatal	1960.10.01–1961.09.30	1592

**Table 2:** Appropriate levels of blood lipids and abnormal stratification standards in Chinese primary arteriosclerotic cardiovascular disease (ASCVD) prevention population.

Level	Total Cholesterol (TC) Concentration	Low-Density Lipoprotein Cholesterol (LDL-C) Concentration	High-Density Lipoprotein Cholesterol (HDL-C) Concentration	Triglyceride (TG) Concentration
Marginally elevated	5.2–<6.2 mmol/L	3.4–<4.1 mmol/L		1.7–<2.3 mmol/L
Elevated	≥6.2 mmol/L	≥4.1 mmol/L		≥2.3 mmol/L
Low			<1.0 mmol/L	

**Table 3:** Characteristics of study population according to Chinese famine exposure.

Name	Unexposed	Postnatal-Exposed	*p*	Prenatal-Exposed	*p*
**Birth Date**	**1962.10–1964.9**	**1959.10–1960.9**		**1960.10–1961.9**	
**N**	**5832**	**2068**		**1592**	
**Level of famine, N (%)**					
Lightly exposed region, N (%)	2383 (40.86)	917 (44.34)	**<0.001**	744 (46.73)	**<0.001**
Moderately exposed region, N (%)	1877 (32.18)	688 (33.27)		515 (32.35)	
Severely exposed region, N (%)	1572 (26.95)	463 (22.39)		333 (20.92)	
Age, years, mean (SD)	48.23 (0.9)	51.61 (0.8)	**<0.001**	50.60 (0.8)	**<0.001**
Male (%)	2365 (40.55)	857 (41.44)	0.315	684 (42.96)	0.063
**Education level**					
Below primary school N (%)	370 (6.34)	188 (9.09)	**<0.001**	128 (8.05)	**<0.001**
Junior high school or below, N (%)	3880 (66.53)	1141 (55.17)		932 (58.52)	
High school or above, N (%)	1582 (27.13)	739 (35.74)		532 (33.44)	
**Marital status**					
Unmarried, N (%)	53 (0.91)	18 (0.87)	0.178	17 (1.07)	0.133
Married, N (%)	5557 (95.28)	1951 (94.34)		1497 (94.03)	
Divorced or widowed, N (%)	222 (3.81)	99 (4.79)		78 (4.91)	
**Per-Capita Annual Income (RMB)**					
Below 10,000, N (%)	2935 (50.32)	1023 (49.46)	0.524	775 (48.65)	0.188
10,000–29,999, N (%)	2484 (42.59)	886 (42.82)		691 (43.38)	
30,000 and above, N (%)	413 (7.09)	160 (7.72)		127 (7.98)	
Inactive, N (%)	5294 (90.78)	1824 (88.2)	**0.004**	1397 (87.77)	**0.002**
Lightly work strength, N (%)	3306 (56.69)	1270 (61.42)	**<0.001**	891 (55.94)	0.588
Control lipid profiles, N (%)	227 (3.89)	100 (4.84)	0.053	55 (3.47)	0.431
BMI, kg/m^2^, mean (SD)	24.43 (3.4)	24.48 (3.4)	0.578	24.41 (3.4)	0.804
Overweight, N (%)	2178 (37.35)	778 (37.61)	0.975	633 (39.77)	0.164
Obesity, N (%)	858 (14.72)	310 (15)		209 (13.15)	
Central obesity, N (%)	1852 (31.75)	700 (33.85)	0.118	540 (33.92)	0.1
TG, mmol/L, mean (SD)	1.52 (1.1)	1.56 (1.1)	0.227	1.58 (1.2)	0.060
TC, mmol/L, mean (SD)	4.67 (1.0)	4.78 (1.0)	**<0.001**	4.73 (1.0)	**0.017**
HDL-C, mmol/L, mean (SD)	1.18 (0.3)	1.20 (0.3)	0.028	1.16 (0.3)	0.052
LDL-C, mmol/L, mean (SD)	2.79 (0.8)	2.87 (0.8)	**<0.001**	2.85 (0.8)	**0.007**
TC/HDL-C, mean (SD)	4.23 (1.5)	4.26 (1.5)	0.380	4.40 (1.7)	**<0.001**
TG/HDL-C, mean (SD)	1.58 (2.0)	1.60 (1.9)	0.751	1.70 (2.2)	**0.027**
AI, mean (SD)	3.23 (1.5)	3.26 (1.5)	0.380	3.40 (1.7)	**<0.001**
